# Switching from alteplase to tenecteplase for stroke thrombolysis: short-term effects on treatment metrics and early functional outcomes

**DOI:** 10.1186/s12883-025-04529-2

**Published:** 2025-12-02

**Authors:** Alexander Seiler, Lea Ingwersen, Naomi Larsen, Olav Jansen, Daniela Berg, Milani Deb-Chatterji

**Affiliations:** 1 Department of Neurology, University Hospital Schleswig-Holstein, Campus Kiel, Kiel, Germany; 2 Department of Radiology and Neuroradiology, University Hospital Schleswig- Holstein, Campus Kiel, Kiel, Germany; 3 Department of Neurology and Neurovascular Center, University Hospital Schleswig-Holstein, Campus Kiel, Arnold-Heller-Straße 3, Kiel, D-24105 Germany

**Keywords:** Acute ischemic stroke, Thrombolysis, Alteplase, Tenecteplase, Treatment metrics, Early functional outcome

## Abstract

**Background:**

Besides its more advantageous pharmacological profile compared to alteplase (rt-PA), increasing evidence suggest a favorable clinical efficacy and safety of tenecteplase (TNK) as alternative thrombolytic agent for the treatment of acute ischemic stroke (AIS). We aimed to analyze the short-term effects of the switch in thrombolytics at our tertiary care stroke center.

**Methods:**

We performed a retrospective analysis of patients receiving intravenous thrombolysis (IVT) for AIS in the time window < 4.5 h, including patients treated with rt-PA (from January 1st to June 30, 2024) and patients treated with TNK (from July 1st to December 31, 2024). Primary and secondary endpoints were the workflow times and the modified Rankin Scale (mRS) at discharge as the early functional outcome. Safety outcomes included any intracerebral hemorrhage (ICH) on follow-up imaging at 24 h, symptomatic (s)ICH and death by day 7.

**Results:**

During the period of 12 months, *n* = 139 patients received IVT in the 4.5 h time window. Median door to needle (DTN) times were significantly shorter for patients treated with TNK (*n* = 74) compared to rt-PA (*n* = 65) (TNK 23 min [IQR 32–17] vs. rt-PA 28 min [IQR 33–22], *p* = 0.047). Patients undergoing endovascular thrombectomy (EVT) after TNK treatment had significantly shorter door to groin (DTG) times in comparison to treatment with rt-PA prior to EVT (*p* = 0.002). No significant differences between the groups were found for early functional outcome (*p* = 0.194 for patients with, *p* = 0.852 for patients without EVT), the rate of sICH (TNK vs. rt-PA: 17.6 vs. 4.2%, *p* = 0.290 with EVT and 3.6 vs. 0%, *p* = 0.507 without EVT) and mortality (TNK vs. rt-PA: 17.6 vs. 4.2%, *p* = 0.290 with EVT and 7.0 vs. 2.4%, *p* = 0.393 without EVT).

**Conclusions:**

Transition from rt-PA to TNK for AIS treatment < 4.5 h after onset at a tertiary care center seems feasible and resulted in shorter onset to treatment durations. Functional outcomes at discharge were comparable between the groups. Rates of ICH, sICH and mortality were numerically higher in the TNK group. Further data are required to assess the clinical efficacy and safety of TNK after the transition from rt-PA in real-world scenarios.

## Introduction

Acute ischemic stroke (AIS) represents the third most frequent cause of death and the major cause of long-term disability in industrial countries [1]. Also after the advent of endovascular thrombectomy (EVT) as effective treatment approach for patients with large-vessel occlusion (LVO), intravenous thrombolysis (IVT) is still the standard of care for the vast majority of AIS patients with a relevant clinical deficit and without contraindications [2]. For several decades, alteplase (rt-PA), which is applied via bolus administration followed by one-hour infusion, used to be the standard thrombolytic agent in stroke centers around the globe [3]. In recent years, tenecteplase (TNK), which is applied as a single bolus, became increasingly distributed as an alternative option for IVT in AIS treatment [3].

From a pharmacological point of view, TNK carries several advantageous properties compared to rt-PA, which include its longer half-life, higher fibrin specificity and increased resistance to plasminogen activator inhibitor-1[4]. These characteristics should lead to an amplified thrombolytic activity, while leading to a less pronounced depletion of fibrinogen compared to rt-PA, resulting in less systemic coagulopathy [4]. Besides these pharmacological advantages, practical aspects make TNK highly attractive for clinical use. Its single-bolus administration may offer advantages in terms of accelerated treatment times and facilitated logistics, especially in cases in which endovascular thrombectomy (EVT) is performed in the primarily treating hospital or after secondary transfer to a thrombectomy-capable center [3, 4]. Several randomized clinical trials demonstrated the non-inferiority of TNK compared to rt-PA concerning efficacy and safety measures [5–11]. Its superiority over rt-PA with regard to achieving excellent functional outcome and reduced disability at 90 days was even suggested by a recent meta-analysis [12].

The official regular approval for TNK for the treatment of AIS in Germany was obtained in February 2024. An expedited recommendation for the use of TNK had been published in the European guidelines in the year before [13] and was mainly based on the results of the AcT trial [5]. However, its use on a larger scale among European stroke units so far remains limited, which may be due to concerns related to the introduction of a newly approved drug in the emergency setting, the substantial effort required to plan and safely guide its implementation in emergency departments and stroke units and pricing issues in the early phase of the commercial distribution of TNK. At our tertiary care stroke center, TNK was implemented as the standard thrombolytic agent for AIS treatment in the time window < 4.5 on the 1 st of July, 2024. Since data on the application of TNK in off-label scenarios with extended or unknown time windows is still limited and its efficacy and safety compared to rt-PA has not been clearly proven so far, the use of TNK was restricted to the time window within 4.5 h after symptom onset/last known well based on inclusion criteria of recent randomized clinical trials [6, 14, 15] and according to our updated local standard operating procedures. Given the implementation of TNK in the middle of the year 2024, two equal time periods can be compared within one year, potentially providing useful real-world insights into the transition from rt-PA to TNK and resulting consequences on local treatment metrics as well as efficacy and safety measures. In this present study, we aimed to evaluate the benefit of TNK compared to rt-PA with regard to a reduction of treatment delays, early functional outcomes and safety measures including the rates of intracerebral hemorrhage (ICH) and mortality at our tertiary care stroke center.

## Materials and methods

This retrospective cohort study covering a period of 12 months was conducted at the Department of Neurology and the integrated academic stroke center at the University Hospital Schleswig-Holstein, Kiel/Germany.

### Study design and data acquisition

After the regular approval of TNK in February 2024, a change of the institutional paradigms and standard operating procedures was planned for the 1 st of July, 2024. From this day on, all AIS patients presenting within the 4.5 h time window from symptom onset who were eligible for IVT were treated with TNK as the thrombolytic agent of choice. All other workflow processes involved in the diagnosis and the treatment of AIS at the emergency department as well as the responsible medical staff members remained unchanged. Patients treated with IVT throughout the year 2024 were identified from our institutional database. Patients receiving IVT within the 4.5 h time window in the first half of 2024 (treated with rt-PA) were compared with the corresponding patient collective in the second half of the year 2024 (treated with TNK). Patients treated with IVT in the extended time window or after unknown symptom onset time were excluded from further analyses.

### Management of acute ischemic stroke

The thrombolytics rt-PA (Boehringer Ingelheim, 0.9 mg/kg) and TNK (Boehringer Ingelheim, 0.25 mg/kg) were administered according to guideline recommendations for AIS management [2, 13] The treatment decision was at the discretion of the treating physicians. IVT with rt-PA was given as a bolus followed by a one-hour infusion (maximum dose 90 mg), TNK was administered as a single intravenous bolus (maximum dose 25 mg). Brain imaging before stroke treatment was performed with CT (Philips Spectral CT 7500) or MRI (Siemens Sola (1.5T) or Vida (3T), Magnetom Series). Treatment decisions for IVT were made according to existing standardized local protocols and SOPs. Large vessel occlusion (LVO) was diagnosed on CT-angiography (CTA) or Time-of-flight MR-angiography (ToF-MRA) and was defined as occlusion of the internal carotid artery (ICA), the middle cerebral artery (MCA) in the first (M1) or second (M2) segment or the basilar artery (BA).

Off-label IVT in patients under oral anticoagulation with vitamin K-antagonists was considered at international normalized ratio (INR) values < 1.7. In patients under direct oral anticoagulants (DOACs), IVT was not considered if the time point of the last DOAC intake could be verified and was within 48 h before admission. In cases with unclear last DOAC intake or unknown antithrombotic medication, anti-Xa activity and Dabigatran plasma levels (Hemoclot assay) were measured and IVT was performed if the results did not suggest effective anticoagulation. Follow-up imaging after AIS treatment was performed at 24 h with routine non-contrast CT imaging. In case of neurological deterioration, follow-up imaging was anticipated. Clinical stroke severity was assessed on the National Institutes of Health Stroke Scale (NIHSS) [16] on admission, after 24 h and at discharge. Recanalization success after EVT was evaluated by a neuroradiologist based on the modified Thrombolysis in Cerebral Infarction (mTICI) score [17], with scores of 2b-3 defined as successful recanalization. Functional outcomes at discharge were assessed using the modified Rankin Scale (mRS) [18] score.

### Clinical variables, outcome and safety parameters

Demographic variables, clinical baseline characteristics and the pre-defined processing times as well as outcome and safety measures were retrieved from the individual patient records generated in the clinical routine during acute ischemic stroke diagnosis and treatment. The demographic variables and baseline characteristics comprised age, sex, pre-stroke mRS, pre-existing vascular risk factors, concomitant antiplatelet drugs or anticoagulants and the stroke etiology. The primary outcome was the improvement of treatment metrics as major indicators of acute stroke workflow efficiency as well as determinants of functional outcome after IVT and EVT. These included the door-to-needle (DTN) time, the needle-to-groin time, the overall door-to-groin (DTG) time, the proportions of patients receiving IVT with a DTN time < 30 min and within the first hour after symptom onset (“golden hour” thrombolysis). Secondary outcomes included the NIHSS at 24 h, the NIHSS and mRS at discharge, the proportions of patients achieving an early excellent (mRS 0–1) and a favorable functional outcome (mRS 0–2) at discharge. As safety outcomes, we used the occurrence of any ICH, the occurrence of symptomatic (s)ICH according to the SITS-MOST criteria [19, 20], parenchymal hematoma type 2 (PH 2) according to the Heidelberg Bleeding Classification [21] and death by day 7.

### Statistical analysis

Categorical variables were described using counts and percentages, while continuous and ordinal variables were described as median and interquartile range (IQR). Proportions were compared between the groups using χ^2^ statistics or Fisher’s exact test, with the corresponding odds ratios (OR) and 95% confidence intervals (CI). Continuous and ordinal variables were compared using the Mann–Whitney U test or *t*-test, depending on the distribution of the data. Apart from univariate analyses with comparisons of outcome parameters between the treatment groups, we performed additional (parsimonious) multivariate linear and binary logistic regression analyses on the entire patient collective to investigate the association of the thrombolytic agent with the continuous outcome parameters DTN and DTG time and the binary outcome parameters excellent and favorable outcome at discharge, sICH and death by day 7. These analyses were used to determine the unstandardized beta-coefficients for the association of the thrombolytic agent (TNK) with the continuous primary outcomes and the odds ratios (OR) for the association of TNK with binary secondary and safety outcomes. Statistical analyses were performed using JASP 0.18.3 (The University of Amsterdam). Apart from processing times, for which a clear hypothesis had been formulated a priori, all tests were 2-sided. The significance level was set to *p* < 0.05.

## Results

### Description of the entire cohort with demographic and clinical baseline characteristics

**Fig. 1:**
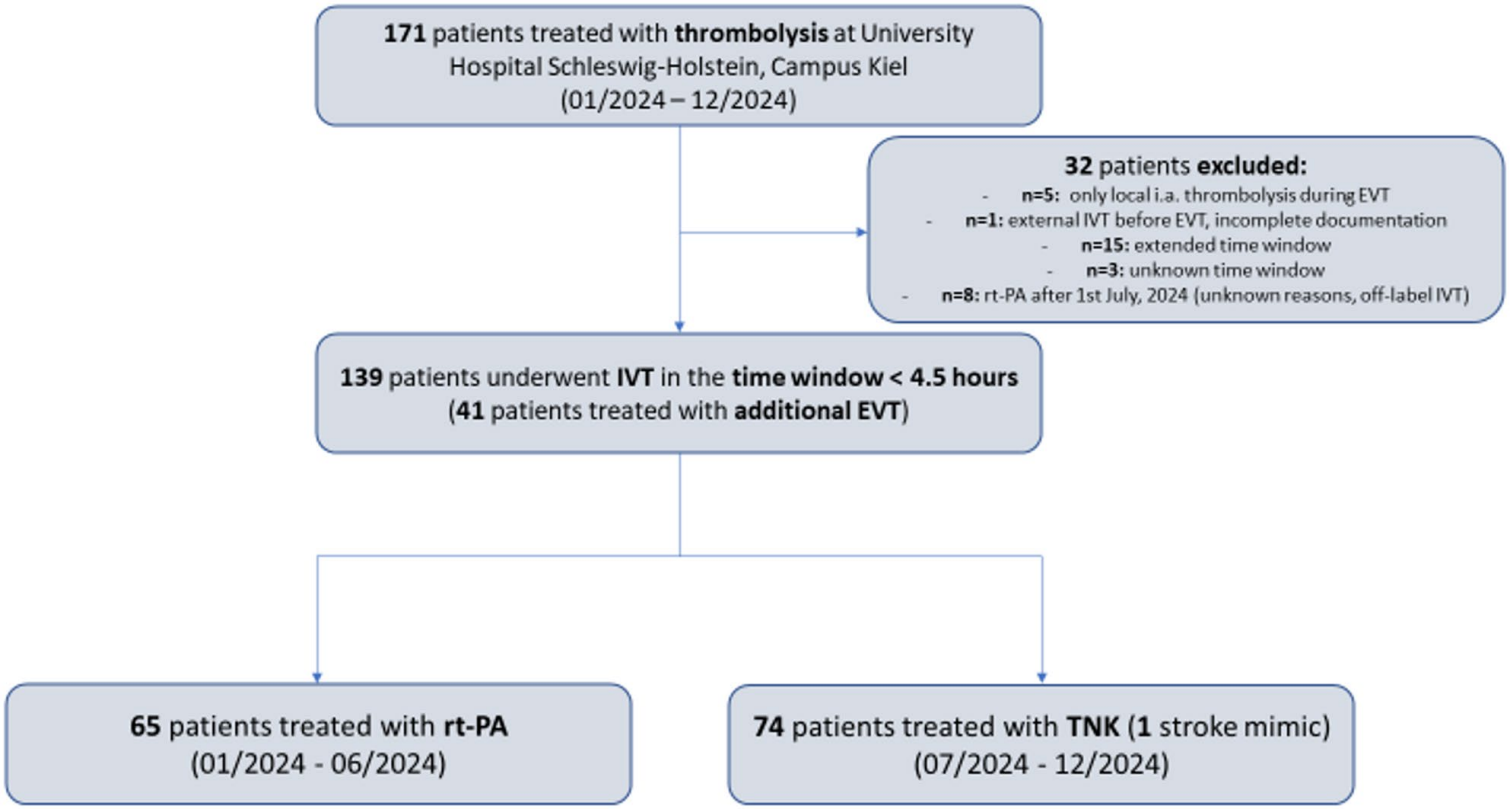
Flowchart summarizing inclusion and exclusion of patients for the retrospective analysis. *N* = 139 patients were included in the final analysis. *IVT* Intravenous thrombolysis, *EVT* Endovascular thrombectomy, *rt-PA* Alteplase, *TNK* Tenecteplase

A total of 171 patients were treated with thrombolytic agents at our institution over a 12-months-period of which *n* = 32 were excluded from further analyses. Reasons for exclusion were mainly the application of exclusive local intraarterial thrombolysis as an individual treatment decision during EVT or IVT in the extended or unknown time window. Of the 32 excluded patients, *n* = 8 had received rt-PA within the 4.5 h time window after the 1 st of July, 2024 for either unknown reasons or in scenarios with off-label thrombolysis. Thus, *n* = 139 patients were retained for the final analysis. Of these *n* = 139 patients, *n* = 65 (46.8%) were treated with rt-PA and *n* = 74 (53.2%) with TNK. One TNK-treated patient was diagnosed with a stroke mimic and was therefore excluded from the analyses of secondary outcomes, while the primary and safety outcomes were included in further analyses also from this patient. A flowchart on patient inclusion and exclusion is provided in Fig. 1.

Both groups were comparable in terms of demographic as well as clinical baseline characteristics, the latter including cardiovascular risk factors and comorbidities, pre-stroke functional status and pre-treatment with antithrombotic medication (Table 1). Furthermore, the distribution of stroke etiologies and baseline clinical stroke severity were comparable in both groups. The proportion of patients presenting with LVO as well as the proportion of patients receiving EVT was higher in the rt-PA compared to the TNK group, with a trend to statistical significance (36.9 vs. 23%, *p* = 0.072). In total, 41 patients underwent thrombectomy after IVT in the 4.5 h time window after symptom onset/last known well, of which *n* = 24 (59%) received rt-PA and *n* = 17 (41%) were treated with TNK. In both groups, successful recanalization was achieved in the vast majority of cases (94.1% in the TNK group vs. 100% in the rt-PA group, *p* = 0.229). Acute stenting during EVT was performed in *n* = 5 patients in both groups (29.4% in the TNK group vs. 20.8% in the rt-PA group, *p* = 0.529). Immediately after acute stenting, *n* = 3 patients (17.6%) in the TNK group received tirofiban for the prevention of early stent thrombosis, compared to *n* = 4 (16.7%) in the rt-PA group (*p* = 1.0). Other antiplatelets were given in the other 2 (11.8%) cases (ASA: *n* = 1, clopidogrel: *n* = 1) of the TNK group, while none of the patients in the rt-PA group (*p* = 0.166) received antiplatelet agents other than tirofiban for the prevention of early stent thrombosis (Table 2). In one case of the EVT subgroup, ASA was accidentally given within 24 h after IVT and thrombectomy. Ninety-eight patients received IVT without additional EVT, of which *n* = 41 (41.8%) received rt-PA and *n* = 57 (58.2%) were treated with TNK. In one case of the non-EVT group, stenting of the ICA was performed shortly after IVT due to high-grade sub-occlusive stenosis. As a concomitant treatment, ASA was initiated immediately after stenting and ticagrelor was added for dual antiplatelet treatment a few days after. Demographic and clinical baseline characteristics of the entire collective are summarized in detail in Table 1.

**Table 1:** Demographic and clinical baseline characteristics for patients treated with rt-PA and patients treated with TNK

	All patients (*n* = 139)	rt-PA group (*n* = 65)	TNK group (*n* = 74)	*p*-value
Age [years] (median (IQR))	73 (81–63)	75 (82–65)	73 (81–61)	0.331
Female sex (n (%))	59 (42.4)	26 (40.0)	33 (44.6)	0.584
Pre-stroke mRS (median (IQR))	1 (2–0)	1 (1–0)	1 (2–0)	0.934
Arterial hypertension n (%))	85 (61.2)	43 (66.2)	42 (56.8)	0.257
Diabetes mellitus (n (%))	27 (19.4)	15 (23.1)	12 (16.2)	0.308
Dyslipidemia (n (%))	91 (65.5)	47 (72.3)	44 (59.5)	0.112
Known atrial fibrillation (n (%))	6 (4.3)	1 (1.5)	5 (5.6)	0.456
Smoking (n (%))	14 (10.1)	9 (13.8)	5 (6.8)	0.166
Coronary artery disease (n (%))	23 (16.5)	10 (15.4)	13 (17.6)	0.730
Chronic heart failure (n (%))	35 (25.2)	14 (21.5)	21 (28.4)	0.354
Stroke etiology (n (%))				
- Cardioembolic - Large-artery disease - Small vessel disease - Other - Unkown	35 (25.2) 28 (20.1) 13 (9.4) 4 (2.9) 59 (42.4)	16 (24.6) 13 (20.0) 8 (12.3) 3 (4.6) 25 (38.5)	19 (25.7) 15 (20.3) 5 (6.8) 1 (1.4) 34 (45.9)	0.571
Prior single antiplatelet treatment (n (%))	37 (26.6)	18 (26.9)	19 (26.4)	0.949
Prior dual antiplatelet treatment (n (%))	4 (2.9)	2 (3.0)	2 (2.8)	1.000
Prior oral anticoagulation (n (%))	2 (1.4)	1 (1.5)	1 (1.4)	1.000
NIHSS admission (median (IQR))	7 (11–4)	7 (11–4)	7 (10–4)	0.473
LVO (n (%))	45 (32.4)	25 (38.5)	20 (27.0)	0.151
Site of LVO^a^ (n (%))				
- Intracranial ICA - MCA M1 - MCA M2 - ICA + MCA M1 - Carotid T - Basilar artery	2 (4.4) 10 (22.3) 16 (35.6) 6 (13.3) 6 (13.3) 5 (11.1)	1 (4.0) 5 (20.0) 9 (36.0) 1 (4.0) 5 (20.0) 4 (16.0)	1 (5.0) 5 (25.0) 7 (35.0) 5 (25.0) 1 (5.0) 1 (5.0)	0.227
EVT (n (%))	41 (29.5)	24 (36.9)	17 (23.0)	0.072
Successful recanalization^b^ (mTICI 2b-3) (n (%))	40 (97.6)	24 (100.0)	16 (94.1)	0.229
Acute stenting during EVT^b^(n (%))	10 (24.4)	5 (20.8)	5 (29.4)	0.529
Tirofiban after stenting within 24 hours^b^(n (%))	7 (17.1)	4 (16.7)	3 (17.6)	1.000
Other antiplatelets after stenting within 24 hours^b^ (n (%))	2 (4.9)	0 (0.0)	2 (11.8)	0.166

*mRS* Modified Rankin Scale, *NIHSS* National Institutes of Health Stroke Scale, *min* Minutes, *IVT* Intravenous thrombolysis, *LVO* Large vessel occlusion, *EVT* Endovascular thrombectomy, *IQR* Interquartile range

^a^Proportions refer to the overall number of patients with LVO in each group

^b^Proportions refer to the number of patients treated by EVT in each group

The interaction of the variables TNK and EVT (TNK*EVT) was evaluated regarding its association with binary secondary and safety outcomes in a binary logistic regression model, in which age, sex, pre-stroke mRS and NIHSS at admission were included as further covariates. The interaction term TNK*EVT was not significantly associated with any of the binary secondary and safety outcomes (all p-values > 0.189). However, due to the higher proportion of patients treated with EVT in the rt-PA group and the different handling of patients undergoing EVT, especially with regard to emergency stenting and early administration of antithrombotics after IVT which are potentially outcome-relevant, secondary and safety outcomes of rt-PA- and TNK-treated patients were also analyzed separately for cases with and without additional EVT and are reported in the subsequent Results section.

### Primary outcomes (treatment metrics) in patients treated with TNK vs. patients treated with rt-PA

The DTN time in cases treated with TNK was significantly shorter compared to rt-PA (23 (32–17) vs. 28 (33–22) minutes, *p* = 0.047). The proportions of patients with “golden hour” IVT and with a DTN time < 30 min were larger in the TNK compared to the rt-PA group, but did not reach statistical significance (*p* = 0.088 and *p* = 0.147). In the subgroup of patients who underwent EVT, there was a shorter needle-to-groin time in the TNK compared to the rt-PA group without reaching statistical significance (*p* = 0.096). The overall DTG time in EVT-treated patients was significantly shorter in the TNK compared to the rt-PA group (61 (63–57) vs. 72 (78–65) minutes, *p* = 0.002) (Table 2).

**Table 2:** Comparison of primary outcomes (treatment metrics) between patients treated with rt-PA and patients treated with TNK

Overall (*n* = 139)	All patients (*n* = 139)	rt-PA group (*n* = 65)	TNK group (*n* = 74)	*p*-value
DTN time [min] (median (IQR))	26 (33–18)	28 (33–22)	23 (32–17)	**0.047**
Golden hour IVT (n (%))	13 (9.4)	3 (4.6)	10 (13.5)	0.088
DTN time < 30 min (n (%))	81 (58.3)	34 (52.3)	47 (63.5)	0.147
**Subgroup of patients with EVT (** ***n*** ** = 41)**	**(** ***n*** ** = 41)**	**(** ***n*** ** = 24)**	**(** ***n*** ** = 17)**	
Needle-to-groin time [min] (median (IQR))	48 (53–41)	49 (53–44)	44.5 (50–36)	0.096
DTG time [min] (median (IQR))	65 (75–59)	71 (78–65)	61 (63–57)	**0.002**

*rt-PA* Alteplase, *TNK* Tenecteplase, *DTN* Door-to-needle, *min* Minutes, *IVT* Intravenous thrombolysis, *EVT* Endovascular thrombectomy, *DTG* Door-to-groin, *IQR* Interquartile range, *EVT* Endovascular thrombectomy

Numbers written in bold indicate statistically significant group comparisons

Multivariate linear regression analysis with the DTN time as dependent variable and the covariates age, sex, pre-stroke mRS, NIHSS at admission and LVO revealed a significant association of the thrombolytic medication (TNK) (unstandardized beta-coefficient − 7.235 (95% CI −13.091 – −0.569), *p* = 0.034) and the NIHSS at admission (standardized beta-coefficient − 0.229 (95% CI −1.457 – −0.096), *p* = 0.026) with the DTN time. After removing the covariate LVO, a multivariate linear regression was also conducted for the DTG time as dependent variable in EVT-treated patients with the other remaining covariates. Here, no significant association could be found between the thrombolytic agent (TNK) and the DTG time in multivariate analysis (unstandardized beta-coefficient − 6.244 (95% CI −16.546–4.057), *p* = 0.226).

### Secondary outcomes (early functional outcomes) in patients treated with TNK vs. patients treated with rt-PA

In the entire patient collective (*n* = 138), there were no significant differences between TNK and rt-PA-treated patients regarding the NIHSS at 24 h (*p* = 0.685) as well as NIHSS and mRS at discharge (*p* = 0.909 and *p* = 0.479). The proportions of patients achieving an excellent outcome were 42.3% in the TNK and 40.0% in the rt-PA group (*p* = 0.769). In the TNK group, *n* = 40 patients (54.8%) achieved a favorable outcome, compared to *n* = 42 patients (64.6%) in the rt-PA group (*n* = 0.241). With regard to the early functional outcomes in the subgroups of patients with (*n* = 41) and without EVT (*n* = 97), there were no significant differences between the TNK and the rt-PA group for NIHSS at 24 h, NIHSS at discharge and mRS at discharge (all p-values > 0.194). The proportions of patients achieving an excellent and favorable functional outcome at discharge in the EVT and the non-EVT subgroup as well as their relation between TNK- and rt-PA-treated patients were similar to the those in the entire patient collective without any significant differences between the IVT groups (Table 3).

**Table 3:** Comparison of secondary outcomes between patients treated with rt-PA and patients treated with TNK

Overall (*n* = 138)	All patients (*n* = 138)	rt-PA group (*n* = 65)	TNK group (*n* = 73)	*p*-value
NIHSS 24 h (median (IQR))	3 (8–1)	3 (6.3–1)	3 (8–1)	0.685
NIHSS discharge (median (IQR))	1 (3–0)	1 (3–1)	2 (3.3–0)	0.909
mRS discharge (median (IQR))	2 (3–1)	2 (3–1)	2 (3–1)	0.479
mRS 0–1 discharge (n (%))	57 (41.3)	26 (40.0)	31 (42.3)	0.769
mRS 0–2 discharge (n (%))	82 (59.4)	42 (64.6)	40 (54.8)	0.241
**Subgroup of patients with EVT (** ***n*** ** = 41)**	**(** ***n*** ** = 41)**	**(** ***n*** ** = 24)**	(***n* = 17)**	
NIHSS 24 h (median (IQR))	5.5 (11–2)	4 (10.5–2)	8 (12–3)	0.323
NIHSS discharge (median (IQR))	2 (3.3–1)	2 (3.5–1)	2 (3–1)	0.724
mRS discharge (median (IQR))	2 (4–1)	2 (3–1)	3 (4–1)	0.194
mRS 0–1 discharge (n (%))	17 (41.5)	10 (41.7)	7 (41.2)	0.975
mRS 0–2 discharge (n (%))	23 (56.1)	15 (62.5)	8 (47.1)	0.326
**Subgroup of patients with EVT (** ***n*** ** = 97)**	**(** ***n*** ** = 97)**	**(** ***n*** ** = 41)**	**(** ***n*** ** = 56)**	
NIHSS 24 h (median (IQR))	3 (5–1)	2 (6–1)	3 (5–1)	0.698
NIHSS discharge (median (IQR))	1 (3–0)	1 (3–0)	2 (3.5–0)	0.950
mRS discharge (median (IQR))	2 (3–1)	2 (3–1)	2 (3–1)	0.852
mRS 0–1 discharge (n (%))	40 (41.2)	16 (39.0)	24 (42.9)	0.705
mRS 0–2 discharge (n (%))	59 (60.8)	27 (65.9)	32 (57.1)	0.385

*rt-PA* Alteplase, *TNK* Tenecteplase, *NIHSS* National Institutes of Health Stroke Scale, *mRS* Modified Rankin Scale, *IQR* Interquartile range, *EVT* Endovascular thrombectomy

Numbers written in bold indicate statistically significant group comparisons

In the multivariate logistic regression analyses for excellent (mRS 0–1) and favorable functional outcome (mRS 0–2) at discharge, the covariates age, pre-stroke mRS, NIHSS at admission and EVT were included. While pre-stroke mRS (*p* < 0.001) and NIHSS at admission (*p* = 0.020 and *p* = 0.040) were significantly associated with excellent and favorable functional outcome in the entire patient collective, the thrombolytic agent (TNK) was not significantly associated with excellent or favorable early functional outcome (OR 1.210 (95% CI 0.531–2.754), *p* = 0.650 and OR 0.577 (95% CI 0.250–1.333), *p* = 0.198) in multivariate analyses. EVT was not significantly associated with excellent or favorable early functional outcome (OR 1.377 (95% CI 0.431–4.399), *p* = 0.589 and OR 0.870 (95% CI 0.274–2.769), *p* = 0.814).

### Safety outcomes (ICH and death by day 7) in patients treated with TNK vs. patients treated with rt-PA

In the entire patient cohort (*n* = 139), any ICH within 24 h occurred in *n* = 10 cases (13.5%) in the TNK vs. *n* = 7 cases (10.8%) in the rt-PA group (*p* = 0.601). Furthermore, sICH (6.8% in the TNK vs. 1.5% in the rt-PA group, *p* = 0.213), PH 2 (4.1% in the TNK vs. 0% in the rt-PA group, *p* = 0.248) and death by day 7 (9.5% in the TNK vs. 3.1% in the rt-PA group, *p* = 0.172) occurred more frequently in the TNK compared to the rt-PA group, without any statistically significant difference (Table 4). In the subgroup of patients who underwent EVT (*n* = 41), any ICH was detected in *n* = 5 cases in each IVT group (29.4% (5/17) in the TNK vs. 20.8% (5/24) in the rt-PA group, *p* = 0.529). Of those, *n* = 3 patients of the TNK group had a sICH, vs. *n* = 1 patient in the rt-PA group (17.6% vs. 4.2%, *p* = 0.290). In the TNK group, *n* = 2 out of 3 sICH-cases occurred after emergency stenting during EVT. One of these patients was treated with tirofiban, and ASA was used as antiplatelet agent in the other case. In patients treated with rt-PA before EVT, the case of sICH occurred after EVT without acute stenting. Since all patients with sICH died, mortality after 7 days was higher in the patient collective treated with TNK compared to rt-PA (17.6% vs. 4.2%, *p* = 0.290) without a statistically significant difference (Table 4). Also in the subgroup of patients without additional EVT after IVT (*n* = 98), both the rate of any ICH and the rate of sICH were higher in the TNK compared to the rt-PA group (8.8% vs. 4.9%, *p* = 0.695 and 3.6% vs. 0%, *p* = 0.507). In one sICH-case of the TNK group, uncontrolled hypertension could be verified as a potential factor explaining the occurrence of sICH. In the case with ICA stenting without thrombectomy after IVT, no ICH or sICH occurred. The patient of the EVT subgroup who had accidentally received ASA after within 24 h after IVT and thrombectomy showed ICH, but not sICH, on follow-up imaging. Mortality within 7 days was higher in the TNK group as compared to the patients treated with rt-PA: *n* = 4 (7.0%) vs. *n* = 1 (2.4%), *p* = 0.393 (Table 4). Only in one of the four TNK-related cases, mortality occurred after sICH.

**Table 4:** Comparison of safety outcomes between patients treated with rt-PA and patients treated with TNK

Overall (*n* = 139)	All patients (*n* = 139)	rt-PA group (*n* = 65)	TNK group (*n* = 74)	*p*-value
Any ICH within 24 h (n (%))	17 (12.2)	7 (10.8)	10 (13.5)	0.601
sICH (n (%))	6 (4.3)	1 (1.5)	5 (6.8)	0.213
Parenchymal hematoma (PH 2)	3 (2.2)	0 (0.0)	3 (4.1)	0.248
Death by day 7 (n (%))	9 (6.5)	2 (3.1)	7 (9.5)	0.172
**Subgroup of patients with EVT (** ***n*** ** = 41)**	**(** ***n*** ** = 41)**	**(** ***n*** ** = 24)**	**(** ***n*** ** = 17)**	
Any ICH within 24 h (n (%))	10 (24.4)	5 (20.8)	5 (29.4)	0.529
sICH (n (%))	4 (9.8)	1 (4.2)	3 (17.6)	0.290
Parenchymal hematoma (PH 2)	2 (4.9)	0 (0.0)	2 (11.8)	0.455
Death by day 7 (n (%))	4 (9.8)	1 (4.2)	3 (17.6)	0.290
**Subgroup of patients with EVT (** ***n*** ** = 98)**	**(** ***n*** ** = 98)**	**(** ***n*** ** = 41)**	**(** ***n*** ** = 57)**	
Any ICH within 24 h (n (%))	7 (7.1)	2 (4.9)	5 (8.8)	0.695
sICH (n (%))	2 (2.1)	0 (0.0)	2 (3.6)	0.507
Parenchymal hematoma (PH 2)	1 (1.0)	0 (0.0)	1 (1.8)	1.000
Death by day 7 (n (%))	5 (5.1)	1 (2.4)	4 (7.0)	0.393

*rt-PA* Alteplase, *TNK* Tenecteplase, *(s)ICH* (symptomatic) intracerebral hemorrhage, * EVT * Endovascular thrombectomy

Numbers written in bold indicate statistically significant group comparisons

In the multivariate logistic regression analyses for sICH with the covariates age, NIHSS at admission, EVT and arterial hypertension, there was a trend to an association of the thrombolytic agent (TNK) with sICH (OR 8.367 (95% CI 0.791–88.473), *p* = 0.077), while EVT was significantly associated with sICH (OR 10.664 (95% CI 1.162–97.874), *p* = 0.036). The multivariate logistic regression analyses for mortality by day 7 with the covariates age, pre-stroke mRS, NIHSS at admission, and EVT revealed a trend for the association of the thrombolytic agent (TNK) with death by day 7 (OR 7.233 (95% CI 0.962–54.369), *p* = 0.055). None of the included covariates was significantly associated with death by day 7.

**Fig. 2:**
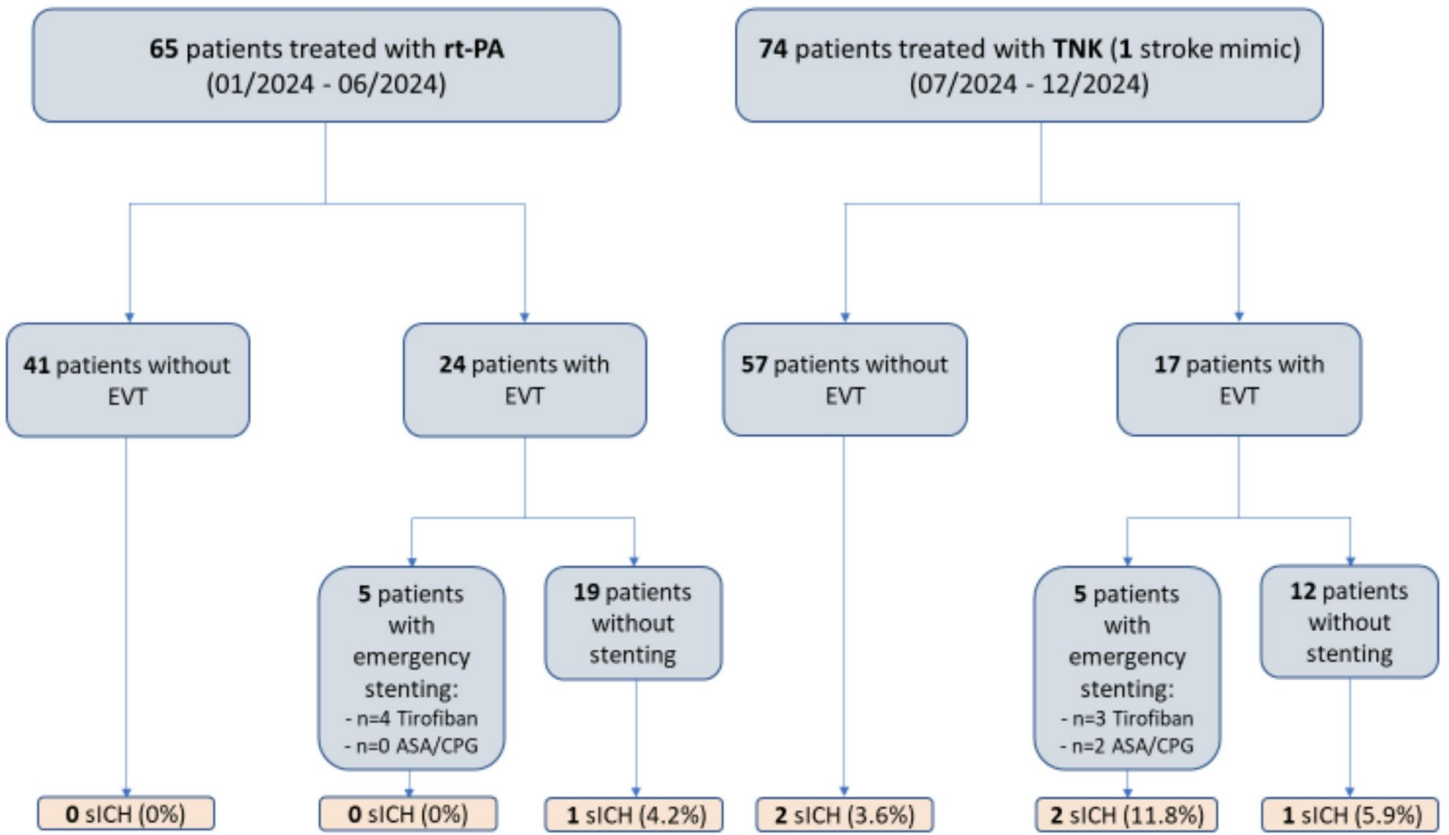
Illustration of further subgrouping of the two main patient groups after stratification according to additional EVT for the analyses of outcome and safety parameters. For each of the illustrated subgroups, the number of patients affected by sICH as well as the corresponding proportions of cases are provided. Percentual rates of sICH refer to the entire subgroup (with or without EVT) in the respective thrombolysis patient group. *rt-PA* Alteplase, *TNK* Tenecteplase, *EVT* Endovascular thrombectomy, *ASA* Acetylsalicylic acid, *CPG * Clopidogrel, *sICH* Symptomatic intracerebral hemorrhage

For the sake of clarity, Fig. 2 summarizes the initial group stratification according to the applied thrombolytic agent and the subgrouping based on whether additional EVT was performed or not. Furthermore, the figure provides information on the proportions of cases with additional emergency stenting and the application of antithrombotics together with the rate of sICH in each of the subgroups.

## Discussion

This study provides real-world data on the transition from rt-PA to TNK as the thrombolytic agent for AIS treatment in the 4.5 h time window at a tertiary care stroke center. Comprising two equally long periods within one year, our study allows for investigating the consequences of the switch in thrombolytics as well as clinical and safety outcomes at otherwise unchanged workflow. In univariate analyses, we found a significant reduction of the DTN and the DTG time in patients undergoing EVT after the switch from rt-PA to TNK (Table 2) and thus a relevant shortening of important treatment metrics, reflecting both the logistic quality and the clinical efficacy of an emergency AIS workflow. In multivariate analysis, at least for the DTN time, a significant association of TNK with shorter treatment times persisted. There were no significant differences in the secondary outcomes between patients treated with TNK and rt-PA, also after subgrouping of the patient collective according to whether additional EVT for AIS treatment was performed or not (Table 3). Also after the adjustment for covariates by multivariate analyses, no significant association between the thrombolytic agent and secondary outcomes was found. Rates of ICH and mortality were numerically higher in all TNK-treated compared to all rt-PA-treated patients including the EVT and non-EVT subgroups, without reaching statistical significance in univariate analyses by group comparisons (Table 4). In multivariate analyses, TNK showed higher odds for sICH and mortality by day 7, however, not reaching statistical significance for both safety outcomes.

Among workflow characteristics in the entire patient collective, the DTN time was significantly shorter in patients treated with TNK as compared to the rt-PA group. The improvement of the DTN time with TNK was observed in some [22, 23], but not in all studies [24] investigating the transition from rt-PA to TNK for stroke thrombolysis. In the study on the transition from rt-PA to TNK from the TETRIS registry [25], the imaging-to-needle time rather than the DTN time was investigated and was significantly shorter in TNK-treated patients. Compared to other studies [22, 23] the DTN times at baseline before the implementation of TNK were relevantly shorter in our study and were further and significantly decreased after the transition. Furthermore, the rate of thrombolysis with a DTN time < 30 min was relatively high in our patient collective [23] and showed a marked, although not significant, increase after the implementation of TNK. Similarly, after the switch to TNK there was a marked increase rate of “golden hour” IVT within the first 60 min after stroke onset, which has been recently demonstrated to increase the probability of a favorable outcome, especially at 90 days after stroke [26] and thus represents an important parameter related to the treatment metrics of AIS management. The significant association of TNK with shorter DTN times could be confirmed in the multivariate analysis after adjustment for relevant demographic and clinical factors. It is plausible to assume that reduction of the DTN time may be driven by a shortening of the imaging-to-needle time as a consequence of simpler and faster medication preparation and administration of TNK compared to rt-PA[23, 25]. In addition, in contrast to previous studies [22, 23] we observed a reduction of the needle-to-groin time and a significant reduction of the overall DTG time after the switch to TNK (Table 2). These results suggest a relevant benefit of the implementation of TNK on the processes involved in our AIS management after the administration of IVT due to facilitated application and logistics with TNK. However, in the multivariate analysis involving the relatively small subgroup of patients treated by EVT (*n* = 41), the association of TNK with shorter DTG times could not be reproduced. Nevertheless, our findings suggest that the transition to TNK may be one effective strategy to reduce also the DTG time and the overall onset-to-treatment time as treatment metrics with potential prognostic relevance. Our sample size may have simply been too limited to demonstrate a significant effect of TNK on the DTG time in multivariate analysis. Despite the favorable effects of TNK on treatment metrics, the more time-consuming administration of rt-PA as a bolus followed by one-hour infusion might theoretically as well be advantageous in special scenarios such as stroke mimics or patients with uncontrolled blood pressure values, since the administration can be stopped at any time when another secondary diagnosis is made or until blood pressure values can be controlled by medication. In our study, only one patient was retrospectively identified with a stroke mimic after IVT with TNK and no detailed documentation was available regarding uncontrolled blood pressure at the time of IVT. Therefore, our study does not allow for investigating potential favorable effects of rt-PA as thrombolytic agent in these special scenarios.

In our study, the clinical benefit with regard to the early functional outcome at discharge was similar in the rt-PA and the TNK group (Table 3). While the proportion of patients achieving an excellent early functional outcome was nearly equal in both groups despite the higher rate of ICH under TNK, the proportion of patients achieving favorable clinical outcome was numerically higher in the group of patients treated with rt-PA, also in the EVT and non-EVT subgroups. Also in multivariate analyses, no significant association was found between TNK as thrombolytic agent and excellent, respectively favorable functional outcome at discharge as pre-defined secondary outcome parameters. These findings are in contrast to the recent results on the switch from rt-PA to TNK from a high-volume stroke center [23], from a stroke reperfusion registry [27] and a regional stroke network [22], which reported higher odds of a favorable functional outcome with TNK after the transition. Results from the TETRIS registry even found a statistically significant difference in the 3-month mRS and significantly higher proportion of patients with a good neurological outcome in favor of TNK [25]. However, the observational study by Koriesh et al.[24] on the switch from rt-PA to TNK described a numerically higher rate of excellent outcome under rt-PA compared to TNK without any significant difference. Thus, on the whole, real-world data on the transition from rt-PA to TNK are still heterogeneous with regard to the clinical benefit of specific thrombolytic agents or advantages of rt-PA over TNK.

Regarding safety outcomes in terms of intracranial bleeding complications, there were no significant differences between the treatment groups before and after the switch (Table 4). However, the rates of both any ICH and sICH, were numerically higher in TNK-treated patients compared to rt-PA, also in the subgroups with and without additional EVT. Similarly, mortality by day 7 was higher in the TNK group, most likely explained by the higher proportion of patients suffering from sICH (Table 4). Multivariate analyses revealed a possible association of TNK with a higher risk of sICH and death by day 7, without statistical significance. Both the rates of any ICH, respectively sICH and the elevated proportion of patients with sICH in the TNK group after the transition are in line with the recently published study from Sekita et al.[23], but could not be reproduced in other studies investigating the switch from rt-PA to TNK [22, 24, 25, 27]. The bleeding rates under TNK found in our study in comparison to rt-PA stand in contrast to reports from a retrospective observational study [28] but are in line with recently published prospective real-world data from the multicenter TETRIS registry [29]. In our study, the rates of any ICH and sICH in the rt-PA group without additional EVT (Table 4) were notably low compared to other studies [24, 27]. Still, the higher ICH rate and the higher mortality in the TNK group may be interpreted as a signal of harm, especially because the multivariate analysis showed a trend for the association of TNK with sICH and mortality when adjustment for EVT as a covariate was performed. Furthermore, the relatively high rate of sICH under TNK in the subgroup of patients treated with EVT (Table 4) deserves further attention. In the subgroup of patients with EVT as a whole, the rates of any ICH and sICH were comparably high, which may be due to the high rate of acute stenting as part of the EVT procedure – especially in the TNK group, in which there was a higher proportion of patients with tandem occlusions of the ICA and MCA M1 segment (Table 1). While the proportion of patients receiving tirofiban as antiplatelet medication was similar in the rt-PA and the TNK group, there was a higher usage of other antiplatelet agents in the TNK group with a trend to statistical significance (Table 1). Of note, 2 out of 3 cases of sICH in the TNK group with additional EVT occurred under early antiplatelet treatment for the prevention of stent thrombosis after emergency stenting as part of the EVT procedure, while one case in both subgroups occurred in a merely embolic LVO without involvement of angioplasty or stenting (Fig. 2). On the whole, we observed a non-significantly higher rate of both any ICH and sICH in patients treated with thrombectomy after IVT with TNK compared to rt-PA. However, upon a more detailed analysis of individual cases with ICH, our results may suggest an elevated bleeding risk especially in patients with LVO due to underlying chronic steno-occlusive disease, in which acute stenting is performed as part of the EVT procedure. It may be speculated that a combined effect of abrupt reperfusion after chronic cerebral hypoperfusion and early initiation of antiplatelet treatment along with the enhanced thrombolytic activity of TNK [4] is responsible for the increased rate of sICH observed in our study cohort treated with TNK prior to EVT. Marnat et al. found a higher rate of any ICH in patients with tandem occlusions and large-artery atherosclerosis etiology treated with TNK compared to rt-PA, but no increased rate of PH or sICH under TNK [30]. In the subgroup of patients with emergent cervical stenting, the rates of both any ICH and sICH were increased under TNK, but not the proportion of patients developing PH and there were no differences between the rt-PA and the TNK group in terms of functional outcome or mortality [30]. However, the sample sizes of the two IVT groups were not well-balanced. Furthermore, that study lacked a dedicated analysis of applied antiplatelet medication early after the intervention for both IVT groups and hemorrhage risk under rt-PA and TNK associated with certain antithrombotic regimens in stented patients could not be assessed in their study [30]. Clinical outcomes and hemorrhagic complications in TNK-treated patients undergoing EVT with emergency stenting and early initiation of antiplatelets should be systematically investigated in future prospective trials. However, considerations regarding signals of higher bleeding risk and mortality under TNK in our study are not restricted to patients treated with EVT (which was independently associated with higher risk of sICH in multivariate analysis), since associations of TNK with sICH and death by day 7 with a trend to statistical significance were identified in the entire patient collective after adjustment for EVT besides other covariates.

### Limitations

This study has several limitations, which have to be addressed. First, it was a retrospective monocentric study and our results should be generalized with caution since an influence of local resources and protocols cannot be excluded. This has to be considered since we found higher rates of ICH and mortality in TNK-treated patients. These were especially marked among patients treated with EVT and additional stenting, which required the early initiation of antiplatelets to prevent stent thrombosis. Furthermore, compared to other studies on the transition from rt-PA to TNK conducted in high-volume stroke centers, our patient collective was relatively small, in particular after further stratification of the treatment groups according to whether additional EVT was performed or not. Therefore, our study was not powered to detect differences in sICH or mortality between the treatment groups. This reduces the informative value of our study and the limited sample sizes of the treatment groups precluded the possibility of performing reliable adjusted comparisons within in subgroups as well as further subgroup analyses. It is plausible to assume that in a larger sample size, the group differences in bleeding rates and mortality would lead to statistically significant effects which are currently masked by the limited sample size. In addition, regarding the positive impact of TNK on processing times and treatment metrics, a potential time effect has to be considered along with other factors potentially influencing workflow efficiency. This has to be considered since the association of TNK with shorter DTG times did not remain statistically significant in the multivariate analysis. We cannot fully exclude that the stroke workflow efficiency at our center may have improved up to a certain extent over time or may be influenced by other factors in individual cases, independently of the implementation of TNK. Finally, our study only included patients who were treated with IVT in the 4.5 h time window and does not allow for drawing any conclusions with regard to the clinical benefit and safety profile of TNK in patients treated in the extended or unknown time window or in other off-label scenarios, including patients on active anticoagulation.

## Conclusions

The transition from rt-PA to TNK for patients receiving IVT in the 4.5 h time window was feasible at a tertiary care center and was associated with shorter treatment times. In terms of the early functional outcomes at discharge, our results do not clearly point towards any favor or disfavor of TNK concerning the clinical benefit of IVT after the switch in thrombolytics in a real-world scenario. In our study, TNK was associated with an overall non-significantly increased rate of ICH and mortality. The increased rates of sICH and mortality in TNK-treated patients were markedly pronounced in the EVT subgroup, especially in cases in which acute stenting was performed during EVT, necessitating the early commencement of antiplatelet medication to prevent stent thrombosis. However, our sample size was too small to draw meaningful conclusions on the risk of ICH after the administration of TNK in this special subgroup of patients. Whereas more real-world data on the transition from rt-PA to TNK are needed and might shortly be available, future prospective randomized trials will be required to investigate the benefit and safety profiles of rt-PA and TNK in patients with tandem occlusions undergoing EVT with emergency stenting.

## Data Availability

The datasets generated and/or analyzed during the current study are not publicly available due to local guidelines on data protection but are available from the corresponding author on reasonable request.

## Abbreviations

AIS: Acute ischemic stroke
IVT: Intravenous thrombolysis
rt-PA: Alteplase
TNK: Tenecteplase
EVT: Endovascular thrombectomy
DTN: Door-to-needle
DTG: Door-to-groin
(s)ICH: Symptomatic intracerebral hemorrhage
NIHSS: National Institutes of Health
mRS: Modified Rankin Scale
LVO: Large-vessel occlusion
ASA: Acetylsalicylic acid
